# Practical Observations on Midwifery

**Published:** 1843-07-01

**Authors:** 


PRACTICAL OBSERVATIONS ON MIDWIFERY; with Cases in Illustration. By *John Ramsbotham*, M.D., &c. &c. Second Edition, revised, in one volume. London, Highley; Churchill. 8vo. pp. 500.

THIS is an excellent work, and well deserves a place in the first rank of practical treatises on the obstetric art.

Among its excellencies—and this is no mean praise—is that it is written in a good style. It is pleasing to read; neither repelling us by diffuseness or tediousness, as is the case with certain other books on midwifery which we could name; nor ever admitting of our laying down the book dissatisfied with what we may have read, as an insufficient development of the subject. It is characterised throughout by the eloquence of simplicity and plain good sense, and it has the inestimable merit of *keeping perpetually to the point*. Midwifery, indeed, when confined within its proper sphere, is essentially a practical art, involving the consideration of little that is hypothetical, or that gives cause for the excursions of fancy; so that discussions on the complicated questions of embryology and other cognate sciences are with justice excluded from a book, the reader of which takes it up solely with the view of deriving guidance in a branch of medical practice and manipulation. The accoucheur, or the midwife is not called upon, like the physiological professor, to indulge in speculations as to the exciting cause of menstruation, the recondite processes of development in the ovum, &c. A clear head, a cool judgment, and a firm purpose, accompanied by an adequate knowledge of the condition of the uterine contents in the later periods of gestation, are—rather than a mind ingeniously fitted for the elucidation of subtle inquiries—the qualities eminently called for in the midwifery-practitioner. A happy method of laying the several heads of obstetric practice before his reader, and the peculiar tact for seizing on the most appropriate cases to illustrate these several heads, will always be the chief, if not the only, literary virtues which the writer on midwifery need aspire to claim. These qualities display themselves conspicuously in the work of Dr. Ramsbotham; and it will, accordingly, constitute in this place our main duty to extract from the treatise before us such passages as exhibit most clearly these distinctive characters.

In a few places, and these are very few indeed—Dr. Ramsbotham has stepped out of the path which he had assigned to himself, into the domain of general anatomy. The opinions of a man like Dr. Ramsbotham, will ever be entitled to respect; but we must say, that in some of these discursive wanderings, he has expressed some, in which, in common with the greater number of physiologists, we may hesitate to concur.

Take, for instance, the passage where, in speaking of the structure of the uterus, he says:—

"Let this viscus be examined with an impartial eye, with an unbiassed mind, either under gravity or unimpregnated, and its muscularity, in the proper sense of the term, must be, I think, with difficulty admitted. * * * Muscular structure consists in a congeries or bundle of fleshy fibres, or filaments, connected together by cellular membrane, and appropriated to motion or action, voluntary or involuntary. Now, if this definition of muscularity be correct, any structure, which does not accord with it in some degree, must be other than muscular. Is there, I would beg to ask, any distinct set, or are there any distinct sets of muscular fibres connected by cellular membrane severally perceptible throughout the whole or any part of the uterine parietes? Or is such a distribution of muscular structure evidently visible in its composition, as appears capable of producing effects equal to those of uterine contraction under the active state of labor? Does the human body offer any instance of muscular structure being for such length of time perfectly quiescent; of its assuming, and acquiring a degree of growth and evolution similar to that of the uterus under a state of impregnation; and, after the performance of certain actions, resuming its pristine state, and appearance without any obvious alteration? If satisfactory answers cannot be given in the affirmative to questions like these, the uterine structure ought not, in my opinion, to be called or considered muscular." 4-5.

We think we need scarcely afford any more space to the combatting this opinion, than such as is required by the following subsequent admission of our author himself, which appears to us difficult to square with the opinion he had previously broached.

"That the uterus under a state of gravidity, does possess strong powers of action, by which its parietes are reduced within a smaller bulk, and by which the capacity of its cavity is diminished to an extent unequalled by any other organ of the human body, is a fact too obvious to be denied." 5.

We have yet to learn that active contractile powers—"strong powers of action," in the decided language of Dr. Ramsbotham—are resident in any other than muscular tissue. But we refer our readers to the whole section on the uterine structure, (p. 367,) for the arguments *pro* and *con*; and we are inclined to think that, after having given it consideration, more of our professional brethren will be disposed with us to dissent from than to agree with Dr. Ramsbotham on this particular subject.

After some useful introductory observations upon the contents of the gravid uterus, and the symptoms of approaching labour, our author enters upon the full consideration of this process; and from this part of his work we extract a few lines, the maxims conveyed in which ought to be ever present to the mind of the medical attendant in the lying-in chamber.

"Throughout the course and management of a common natural labor, the assistance of the accoucheur is seldom wanted till the expulsion of the child is at hand: he has merely to superintend the process; to take care that all the natural changes are duly and timely performed; and to provide against any avoidable injury which neglect might occasion. By untimely and officious interference, the whole process is frequently thrown into derangement and confusion; the use of instrumental means, is thus called for towards its close, to ensure the welfare of the mother: whereas, in all probability, had a different line of conduct been pursued, a natural and safe termination would have resulted." 25.

In his section on the general management of the placenta, Dr. Ramsbotham inculcates the following judicious method of proceeding, and points out the inferences which it is the means of establishing.

"After the separation of the child, the hand of the accoucheur must always be applied upon the lower part of the abdomen, with the intention of ascertaining the actual condition of the uterus, and the degree of contraction it has already undergone; for every other consideration is now of minor importance in comparison with that of uterine contraction. This simple proceeding ought never to be omitted: it enables us to judge of the probable safety of our patient, and to give those satisfactory assurances which ever prove so pleasing: it warns us of threatened mischief, and empowers us to take timely steps to avert it: it is also the surest means of detecting the presence of a second child. By the state in which the uterine tumor is now found under the hand must the practice be regulated. * * * * If the uterus be now found low in the abdomen, or in the pelvis; if it be firm, well contracted, and small in bulk, the safety of the woman is pretty well assured. If, on the contrary, the uterus remain high, if it be flaccid, ill-contracted, and large in size, without the presence of a second child, some threatening of mischief attaches to such symptoms, of which the accoucheur is forewarned." 27-30.

With respect to the *period of time* which may be suffered to elapse before the placenta, if retained, should be withdrawn artificially, our author makes observations which are as remarkable for the sound judgment that has dictated them, as they are characteristic of having been the result of grave and protracted deliberation, and a careful weighing of all the circumstances capable of producing retention of the placenta.

"Though the placenta may be withdrawn at pleasure, it may be a question of policy, whether it ought to be withdrawn *immediately*.
* * * "But let us suppose that the placenta still remains entirely within the uterine cavity; that there is no tendency to a return of uterine action; that the uterine tumor continues high, large, and flaccid; what length of time are we justly authorized to wait before some decided steps should be taken for its removal out of the uterus? * * * I am ready to acknowledge that there is great difficulty in fixing the precise time for acting. On this important point, the accoucheur must rather be guided by the respective circumstances of the case, as they arise; by the general state of the patient; by the feel of the uterine tumor; by the quantity of sanguineous discharge, and its effects; and by the nature and length of the preceding labor, than by simple attention to lapse of time.

"It will rarely be necessary to exceed two hours before recourse should be had to this proceding: more frequently its necessity will be obvious before the expiration of this time; indeed, I think, on an average of cases, it will be found that, if the placenta be not thrown off by natural means within one hour from the birth of the child, it is detained by some unusual cause. If hæmorrhage or other pressing symptom should suddenly intervene, an earlier removal will be required; otherwise every thing like hurry or haste ought carefully to be avoided." 31-37.

We must not allow ourselves to mutilate the admirable chapter on adhesion of the placenta, (pp. 47-74,) which we, therefore, reluctantly pass over without extract or comment. But we most cordially concur with Dr. Dewees, the editor of the American edition of this work, that the whole of this chapter "shows a master-hand:" and, indeed, we should be inclined to point to it as *one* of the best—if not eminently the best—of the divisions of the work.

Dr. Ramsbotham has before justly deprecated any attempt to interfere with the progress of natural labour; and he reiterates his caution and his protest in treating of cases in which the process of parturition is being performed in a normal although protracted manner. Thus, he says—

"Any attempts to hasten the labor by forcing the pains, by irritating the os-uteri, by injudiciously rupturing the membranes, by forcibly dilating the external parts or vagina, or by other artifices, under the specious pretence of doing something for the benefit of the patient, are equally reprehensible and injurious. And here I must beg to remark, that I cannot give my sanction to those experimental applications of active substances to the os-uteri, with the view of producing its relaxation, which are made and recommended to be made even by men of experience. * * *

"It appears to me, that labor-pains (properly so called) do form, and were intended by the Great Author of Nature, for the wisest purposes, to form, a constituent part of the act of child-birth; that they are inseparably attached to it as a cause; that they are merely an external evidence of the presence and progress of those powers by which the process is finally to be terminated, but without a due degree of activity in which it must be prolonged; and that they ought not, generally speaking, or on the application of a general principle, to be counteracted. I am certain that they ought not to be entirely suspended: I have my doubts whether, except in very rare instances, any attempt should even be made to palliate them. Pain is certainly an evil, and is universally deprecated as an evil; it seems always highly desirable, to get rid of it as soon as we can; but labor-pain is established to bring about the happiest results. It is, then, one of those necessary evils to which we must patiently submit, within reasonable bounds." 127-129.

And as respects the injudicious, but too frequent employment of opiates besides the degree of benefit which may be legitimately derived from this remedy, he adds—

"The injurious effects of opiates are not simply confined to the retardation or disturbance of labor previous to the expulsion of the child; they are continued to, and exerted upon, that uterine power, by which the placenta ought to be separated and excluded; in default of which it is detained within the uterus, and thus flooding and other mischiefs ensue, from the same source. * * *

"When uterine action has been prematurely and violently established, a little relief has sometimes been procured by repeated small doses at short intervals; after which the labor has proceeded more favorably. But when a truce is thus obtained, their use should be discontinued." 130-131.

Our limits oblige us to pass with very brief notice over the chapters devoted to Preternatural Labour and Uterine Hæmorrhage. But one passage we find it impossible to omit extracting. The moral principles which are here and elsewhere enforced are so correct that, in deciding upon the weighty and jurisprudential questions which arise under the management of difficult cases, we cannot adopt a safer guide to our determinations than the author before us. Thus, on the subject of cephalotomy, we find it remarked,—

"A dreadful degree of responsibility attaches to the accoucheur in every instance of perforation of the head. The operation can never be a matter of choice: it is one of imperious necessity, to which he is impelled, with whatever reluctance, by the strictest sense of professional duty. If the child be alive when the head is perforated, its life is certainly destroyed, and infanticide is committed; yet, for the reason just stated, viz. that the act is not a matter of choice but of necessity to save the mother, it is a justifiable act, and ceases to be a criminal one. Should we even possess satisfactory proof that the child is dead in utero, as, for instance, under a case of simple, but lingering labor, with the funis below the head, devoid of pulsation; though no violence would be offered to the child by the perforation of the head, we ought to abstain from an unnecessary resort to it, even for the sake of appearances alone. But if, in such case, the labor should become protracted, rather than allow the mother to run any risk under the efforts of natural expulsion, I would not hesitate to lessen the head, especially if there existed the least relative disproportion." 177.

No moralist can cavil at this!

The following remarks, also, which chiefly relate to the practice when hæmorrhage occurs from implantation of the placenta over the os-uteri, deserve to be inserted here entire.

"Let me offer an urgent caution against a mode of practice I have sometimes seen pursued under a rigid state of the os uteri. I allude to an attempt to forcibly dilate it, by passing two or more fingers within its orifice, without any intention of immediately introducing the entire hand. Such an act can answer no good purpose: it can only produce a greater portion of placental separation, with its subsequent alarming consequence. * * * When the operation of turning is determined upon, and is once commenced, the difficulties to be encountered in that proceeding are to be met with fortitude, and a cautious perseverance to its termination. The left hand, formed into a conical shape, is to be introduced into the vagina, then gradually through the os uteri into the uterus itself. At the moment of dilating and passing the os uteri, the hæmorrhage is tremendously increased, and if at this moment, from alarm or other cause, the operator should be induced to withdraw his hand, the consequences will be frightful and serious indeed. When he has got thus far in the operation, therefore, he must proceed onward at all risks. If the os uteri be found but little dilated, and be somewhat rigid, it must be carefully and gradually opened by one or more fingers, afterwards by the thicker part of the hand, until the entire hand can be gradually slided within the uterine cavity. The route which the hand must then take will be decided by the occurrences of the moment. But it will generally be found more easy to pass the hand by the side of the placenta, than to penetrate its substance. After entering the uterus, the hand ruptures the membranes, seizes hold of one foot, or both feet (if they can be readily met with,) and brings down the breech through the os uteri, the pressure of which upon the bleeding vessels tends materially at this moment to check the discharge. Having gained these advantages, the operator now procures a little respite from action; and of this interval it is desirable to take advantage carefully to inspect the situation of the woman. If the loss already sustained shall have brought on syncope, or excessive faintness, recourse must be had to stimulants, which have been previously got ready at hand; but if it should not seem to have made much impression, stimulants may be dispensed with.

"When I have been obliged to have recourse to a forced delivery by turning, under a state of great exhaustion, I have frequently fancied, that the shock inflicted upon the nervous system by the violence of the operation has greatly increased the danger of the woman, and has sometimes hastened a fatal result. In reflecting upon this presumption, in cases of sudden depression under a placental presentation, it has seemed to me desirable, if possible, to obtain a truce from the flooding before delivery is attempted, that the system may somewhat rally from its preceding effects. I have therefore thought, that if, in these desperate cases, by any gentle means, the liquor amnii could be discharged, without inducing a greater degree of placental separation, some advantage would be derived from uterine contraction, and the violence of the discharge would be thereby checked. I must however in candour declare, that I have not had an opportunity of realizing the practical effect of this suggestion since it occurred to my mind; I offer it therefore merely as an object of future consideration. The method I would propose is, to penetrate the centre of the placenta by a perforator, or other sharply-pointed instrument, and allow the liquor amnii to run off. If the discharge should be thereby checked, delivery may be put off for a short time; but if the discharge should continue afterwards, delivery must not be delayed. Let it be clearly understood, however, that this act will not supersede the necessity of delivery sooner or later, and that it will cause some loss of the child's blood from the laceration of the placental vessels.

"I have repeatedly remarked, that among those cases which have terminated fatally, in several of them that event has seemed to me to be hastened by too quick an extraction of the child; by too sudden evacuation of the uterine contents. If the hand in turning be allowed to enter the uterus without resistance, and if, after it is in complete possession of the cavity, no contractile effort be perceptible in the parietes, the extraction of the child should be very gradually performed. When the breech is brought down, its pressure generally suspends the discharge. When this is the case, there can be no immediate necessity for the quick extraction of the body and head; and I feel perfectly satisfied, that by such a mode of proceeding, much injury is occasionally done to the woman. But on this point, as on many others, the practice must be regulated by the state of the woman, and that of the child, under due discretion and judgment." 295-302.

Fortunately, cases of this nature demanding so large an exercise of judgment and firmness on the part of the obstetric practitioner are comparatively rare.

The convulsions that occasionally take place in the puerperal state are described in a really graphic manner (pp. 316-19); and Dr. Ramsbotham seems strongly inclined to join M. Andral in attributing a greater tendency to their occurrence to a peculiar electrical condition of the atmosphere. They are certainly more often observed to happen in thundery weather. Dr. Ramsbotham advises for their treatment,—

"The abstraction of blood, and the free evacuation of the bowels, the constant application of cold evaporating fluids to the whole surface of the head, or the local affusion of a stream of cold water upon the vertex." 326.

The writer of the present article suggests, as a more manageable mode than any other hitherto proposed of obtaining the salutary effects of refrigeration—the use of the *cold pillow*, recommended by his preceptor the late Professor Davis. This apparatus, which is composed simply of a large bullock's bladder, about three-parts filled with cold water, and is in general readily attainable, has the advantage of being continually applicable to the vertex of the patient, without the discomfort that attends repeated cold affusions, and with the advantage of being changed with ease, as often as the water contained loses its necessary coldness. In more than one case, the writer has had the happiness of bringing labor with puerperal convulsions to a successful termination, a result which he has in no small degree laid to the instrumentality of the "*cold pillow*."

Many useful practical remarks are comprised in the chapters on Rupture and Retroversion of the Uterus.

The necessity for attending to the condition of the bladder and rectum, and regularly evacuating their contents (particularly those of the former organ) during pregnancy, is strongly insisted on; and many distressing circumstances are shown to have arisen from a non-attendance to these peremptory duties alone. While speaking of rupture of the uterus, our author remarks.

"In all cases of this accident, there is a narrowness if not an absolute deformity of the pelvis, so that perforation of the head becomes, too commonly, indispensably necessary to the delivery. But if the presenting part of the child shall have retreated from the situation which it had previously occupied, so that a considerable portion of the child has escaped into the abdominal cavity, delivery must be effected by the introduction of the hand, and extraction by the feet." 428.

*Speedy Delivery* is proved by Dr. Ramsbotham to be decidedly the indication of treatment in the event of this perilous accident; and by resorting to it he has himself been enabled to save the life of the patient, and even procure her restoration to health, in two out of three successful cases of this nature, which are recorded at the end of the work.

In alluding to the doctrine of superfœtation, our author asserts, p. 391, "an enlightened physiology has nearly exploded the idea of that occurrence,"—but this is an error, if we are to entertain the theories of Raciborski, Drs. Power, Laycock, &c. which contribute to show that superfœtation is not only a very possible event, but probable as well—and offer for it a satisfactory rationale.

We could well have pardoned Dr. Ramsbotham, for pronouncing *ex cathedra* on this and other critical points, upon which no clear light has been thrown till lately. But his objections are always gentle: he has no offensive dogmatism; and with the advantage of obstetric medicine always at heart, he frankly acknowledges when he has been misled.

Dr. Ramsbotham's treatise does not extend to the management of either the mother or child after the completion of delivery. Neither is it furnished with plates of any description; but the principles and practice inculcated are illustrated by upwards of 170 well-selected cases. Altogether, if this work be less copious and comprehensive than some others of its class, it is still of high value; and, not only as a companion to other works, but for its intrinsic merits, it ought to have a place in every public and private medical library.

